# Osteoarthritis of zygapophysial joints as a cause of back pain and neck pain: a scoping review

**DOI:** 10.1093/pm/pnae036

**Published:** 2024-05-03

**Authors:** Nikolai Bogduk, John MacVicar

**Affiliations:** School of Medicine and Public Health, University of Newcastle, Newcastle, NSW, Australia; Southern Rehab, Christchurch, New Zealand

**Keywords:** lumbar, cervical, zygapophysial, facet joint, osteoarthritis, degeneration, pain

## Abstract

**Background:**

Zygapophysial joints (Z joints) can be a source of back pain and of neck pain, but the cause of pain is not known. Some authors attribute the pain to osteoarthritis but without citing evidence.

**Objectives:**

The present review was undertaken to determine if there was sufficient evidence of association between spinal pain and osteoarthritis of Z joints to justify osteoarthritis being held to be the cause of pain. The null hypothesis was that osteoarthritis of Z joints does not cause back pain or neck pain.

**Eligibility criteria:**

Relevant studies were ones that provided primary data on the association between pain and osteoarthritis of Z joints. These could be population studies, diagnostic studies, or case-control studies.

**Sources of evidence:**

The database of PubMed was searched using the terms: Lumbar or cervical, zygapophysial or facet, pain, and osteoarthritis or degeneration or degenerative.

**Charting methods:**

Data pertinent to the research question were extracted from original articles and tabulated for reporting. Odds ratios for associations were calculated, as were the prevalence rates of osteoarthritis in subjects with pain, and conversely the prevalence rates of pain in subjects with osteoarthritis.

**Results:**

The searches retrieved 11 population studies, 4 diagnostic studies, and 3 cases control studies. No study showed any positive association between osteoarthritis of Z joints and pain. All studies found pain to be independent of the presence or severity of osteoarthritis. Osteoarthritis was as common in subjects with no pain as in subjects with pain. The null hypothesis was not refuted.

**Conclusion:**

The published evidence does not support the belief that osteoarthritis causes Z joint pain. All the evidence contradicts this belief.

## Introduction

Zygapophysial joints (Z joints), also referred to as “facet” joints, have increasingly been recognized as possible sources of chronic neck pain and chronic low back pain. Using controlled, diagnostic blocks, studies have shown that the source of pain can be traced to one or more Z joints in 36%-60% of patients with chronic neck pain, particularly after trauma,^1–5^ and in 60% of patients with probable cervicogenic headache.^6^ In patients with chronic low back pain, the prevalence of Z joint pain ranges from 15% to 45%, depending on the criteria used for a positive response to diagnostic blocks.^7–9^ Once diagnosed, Z joint pain can be successfully treated by radiofrequency medial branch coagulation.^10^^,^^11^

However, although Z joints have been established as a source of spinal pain, the pathology responsible for that pain has been elusive. Overt lesions, such as infection,^12^^,^^13^ gout or crystal arthropathy,^14–16^ pigmented villonodular synovitis,^17^ giant cell tumors,^18^ and other neoplasms^19^ are rare, having been recorded only in case reports.

In contrast, Z joints are commonly affected by osteoarthritis.^20^ This coincidence has given rise to the contention that osteoarthritis is a common cause of Z joint pain. This contention is attractive to physicians because osteoarthritis can be detected on plain radiographs, computerized tomography (CT) scans, or magnetic resonance images (MRI); and finding this pathology provides a convenient explanation for why the joint is painful. This contention is also attractive to compensation insurers for, if the patient’s pain can be attributed to naturally occurring osteoarthritis, it can avoid being attributed to a compensable injury.

Belief in this contention can adversely affect the practice of pain medicine. If denied coverage under compensation insurance, patients might not be able to access the services required to diagnose the source of their pain, and to have it relieved.

Despite these adverse effects, this contention continues to be promulgated, both in the pain literature, and in medicolegal proceedings. Curiously, however, those who promote the belief do not cite any evidence that supports it. Therefore, in order to test this contention, a review was undertaken to collate and assess the available evidence on osteoarthritis as a cause of Z joint pain.

## Methods

Although guidelines are available for conducting systematic reviews of the treatment of pain,^21^ equivalent guidelines have not been formulated for reviewing causes of pain. As a suitable surrogate, the present review adopted the principles of scoping reviews.^22^ These principles ask that the review should identify and map the available evidence, in terms of the volume of literature available, the types of evidence, the methods used to produce that evidence, and any gaps in the evidence. They also stipulate that an a priori protocol be followed for systematic searching that is transparent and reproducible, with measures to reduce error and to increase reliability; and that data be presented in a structured way.

For the present review, the literature was identified by interrogating the database of PubMed using various combinations the terms: Lumbar or cervical, with facet or zygapophysial or zygapophyseal, and osteoarthritis and/or pain. Searches were also conducted using degenerative or degeneration instead of osteoarthritis. Searches were not restricted to any date of publication. Publications in any language were entertained.

The titles and abstracts of publications identified by these searches were inspected to identify those that might provide empirical evidence that related osteoarthritis of Z joints to pain. Full versions of such publications were retrieved for further assessment.

Several actions were taken to check the fidelity and reliability of the searches undertaken. The database of Embase was interrogated using the same search terms to see if any additional articles could be found. The reference lists of previously published review articles were consulted to see if they contained any articles not identified by the searches. The articles retrieved were compared with the private libraries of the present authors. Searches were first conducted in September 2023, and were repeated in January 2024.

In order to be included in the review, articles had to be primary studies that provided original data on the association between pain and osteoarthritis of Z joints. Not included were articles that simply offered opinions or assertions. Three main types of studies were anticipated for inclusion: Population studies, diagnostic studies, and case-control studies.

Population studies compare the presence and absence of osteoarthritis with the presence and absence of pain in a sample of patients drawn from the general community. Odds ratios can be calculated to compare the odds that osteoarthritis occurs in patients with pain, and the odds that it occurs in patients with no pain. An odds ratio becomes statistically significant if its 95% CIs do not overlap a value of 1.0.^23^

A deeper consideration involves examining the prevalence rates of osteoarthritis and pain in population studies. For osteoarthritis of the Z joints to be incriminated as the cause of pain, the prevalence of pain should be substantially greater in individuals affected by osteoarthritis than in unaffected individuals; and the prevalence of osteoarthritis should be substantially greater in individuals with pain than in those with no pain. Unless these criteria are satisfied the distribution of osteoarthritis is essentially random, and the cause of pain cannot be distinguished from some other (unknown) cause of pain that equally affects individuals with and without osteoarthritis. The statistical threshold for considering that one feature is more common than another is that the 95% CIs of their prevalence rates do not overlap. Conversely, if those CIs overlap, there is no difference between rates.

Population studies were included in the present review if they reported data on the presence and absence of osteoarthritis and the presence and absence of pain in the relevant region of the spine. For purposes of reporting and analysis, raw data on these variables were extracted from the text, tables, or figures of eligible studies. Those data were then combined into tables. Odds ratios were calculated using VassarStats (http://vassarstats.net). Prevalence rates were calculated using Epitools (https://epitools.ausvet.com.au).

Diagnostic studies are ones in which a joint affected by osteoarthritis is tested for being a cause of pain by anesthetizing it to see if doing so relieves the patient’s pain. In order for osteoarthritis to be impugned as a cause of pain in general, diagnostic blocks of affected joints in a series of patients should consistently relieve their pain, or do so in a substantial proportion of cases. In such studies, the blocks must be controlled, in order reduce the number of false-positive responses. If responses to blocks are false-positive, any conclusions based on those responses will also be false. Controls are less crucial if results are negative. In principle, it seems pointless and ethically questionable to proceed with further blocks when the first block is negative, for an inordinate number of additional blocks would be required to show conclusively that the first response was false-negative.^24^ (According to the binomial theorem the number blocks required (*n*) is given by ^*n*^C_1_/2^*n*^<0.05, which amounts to at least 8 blocks.)

Diagnostic studies were included in the present review if they reported the numbers of patients whose pain was relieved or not relieved when joints affected and not affected by osteoarthritis were anesthetized. Studies with purportedly positive results were regarded as more compelling if they reported the prevalence of complete relief of pain as opposed to partial relief. Although some physicians believe that partial relief of pain means that the joint is partially responsible for the patient’s pain,^25^ that interpretation is specious unless and until the source of the remnant pain is established. Unless that is done, a partial response to blocks cannot be distinguished from some form of placebo response or hedging on the part of the patient.^26^

When studies provided raw data, those data were extracted and summarized in Tables. Odds ratios and their 95% CIs were calculated. When a study did not provide raw data, its summary data were copied and tabulated for reporting.

In case-control studies, the presence and absence of osteoarthritis are compared between patients who present with pain and subjects with no pain. The latter may be individuals from the general community who volunteer to undergo imaging, or they may be patients who have undergone spine imaging for conditions other than spinal pain.

Case-control studies were included in the present review if they satisfied the same eligibility criteria that were applied to population studies. Data were extracted, reported, and analyzed in the same way as for population studies, using odds ratios and prevalence rates.

## Results

### Retrieval

For lumbar Z joint osteoarthritis, a search using the terms: Lumbar AND facet AND osteoarthritis, yielded 315 titles, of which 11 were retrieved. These were 5 primary studies,^27–31^ 4 reviews,^32–35^ and 2 teaching articles.^36^^,^^37^ Not retrieved were articles that addressed other topics, such as treatment, diagnostic blocks, or imaging, which did not refer to any relationship between osteoarthritis and pain.

An additional search, using the terms lumbar AND facet AND degenerative or degeneration yielded 1532 titles. Of these were 2 eligible studies that had already been retrieved^32^^,^^34^ and 5 additional articles^38–42^ not found by the previous search. As shown in Table 1, other combinations of search terms yielded the same, or some of the same, articles, but no additional articles. A search of Embase found no additional articles.

**Table 1.** The number of articles listed in searches using the combinations of terms shown, and the number of those articles retrieved for inclusion in the review.

	Articles		
		Retrieved	
Search terms	Listed	Same	New
Lumbar			
AND facet AND osteoarthritis	315	11	
AND facet AND osteoarthritis AND pain	106	11	
AND zygapophysial AND osteoarthritis AND pain	4		
AND facet AND degeneration or degenerated	1532	2	5
References in previously published reviews			2
Personal libraries of the present authors			2
Cervical			
AND facet AND osteoarthritis	43	3	
AND facet AND osteoarthritis AND pain	43	3	
AND zygapophysial AND osteoarthritis AND pain	4		
AND zygapophyseal AND osteoarthritis AND pain	23	2	
AND facet AND degeneration or degenerative	284	1	1
Personal libraries of the present authors			3

An older review^33^ referred to one population study^43^ and one diagnostic study^44^ that were not found by the present searches, but which were otherwise known to the present authors. The population study^43^ focused on so-called degenerative changes in lumbar intervertebral discs. Although this study referred to Z joints, it provided little usable information about them; but greater detail was provided in an online supplement to a review article on degenerative changes,^45^ with which the present authors were already familiar.

The diagnostic study^44^ was not found because it was cast as a study of the predictive utility of CT scanning, and did not mention osteoarthritis in its title, abstract, or text. It referred only to “changes” seen on imaging, such as joint narrowing and sclerosis, without attributing these to osteoarthritis or degeneration. This article was already known to the present authors because the senior author (N.B.) was an author of that article.

Two other primary studies were not found by any of the searches, and were not included in any earlier reviews, but they had been acquired by the senior author (N.B.) some 45 years ago. Strangely, one is not indexed in PubMed,^46^ despite 9 contemporary, companion studies by the same author being indexed. The other^47^ is indexed in PubMed but without an abstract, and its title does not mention facet or Z joints. It primarily dealt with osteoarthritis and rheumatoid arthritis of the appendicular skeleton, but contained data on “nonrheumatoid” arthritis of the cervical and lumbar Z joints, although it referred to them by the British term: Apophyseal. Prompted by this language difference, a supplementary search of PubMed was conducted using the terms: Apophyseal AND osteoarthritis, but this yielded no studies of interest.

For osteoarthritis of the cervical Z joints, a search using the terms: Cervical AND facet AND osteoarthritis, yielded the greatest number of articles, of which 3 were retrieved. One was a review^33^ and 2 were primary studies.^48^^,^^49^ A search using the terms cervical AND facet AND degenerative or degeneration revealed one of the studies already retrieved^49^ and an additional study^39^ that had already been retrieved in the searches about lumbar Z joints. Other combinations of search terms had the same or lesser yields, but did not provide any articles additional to those already retrieved (Table 1).

Not provided by the searches were 3 older studies held in the personal libraries of the present authors. One was a population study that provided data on cervical Z joints, but which had already been retrieved for data on lumbar Z joints.^47^ The second was a case-control study, indexed in PubMed but without an abstract; its title did not mention Z joints, and its text referred to them only as “articular processes.”^50^ The third was a case-control study.^51^ Neither the title nor the text of this article mentioned facet joints, Z joints, or apophyseal joints; the text referred to them only as “severe joint changes.”

### Population studies lumbar

Two of the 9 population studies of Z joint osteoarthritis were not particularly helpful. In the study of Savage et al.,^42^ MRIs were obtained in 149 male volunteers as part of a study of occupational health. The study focused primarily on disc degeneration and disc herniation. Although osteoarthritis was not explicitly mentioned, facet hypertrophy was, but in only 11 subjects, which is too few for a valid statistical interrogation.

The study of Tiwari et al.^39^ performed CT scans on a sample of subjects referred to as living in the mountains. The authors claimed to have compared the prevalence of osteoarthritis at each cervical, thoracic, and lumbar segmental level in patients with and without pain. However, the numbers reported in their Tables of data were not prevalence rates; they were the absolute numbers of joints affected by osteoarthritis. These numbers had not been divided by the numbers of patients with pain or without pain at cervical, thoracic, and lumbar levels. Nowhere in the published article were these latter numbers reported. So, the actual prevalence rates could not be calculated and compared, even post hoc.

Other population studies were more robust. Six provided evidence on lumbar Z joints,^27–30^,43,46 and one provided data on both lumbar and cervical Z joints.^47^

The study of Lawrence et al.^47^ drew its subjects from a large X-ray survey of 1098 men and 1198 women, designed to study various clinical and demographic features of arthritis in general. After accounting for subjects with rheumatoid arthritis, its sample consisted of 483 men and 494 women who underwent radiography of the lumbar spine.

The study of Magora and Schwartz^46^ drew its subjects from a large survey conducted to investigate the relationships between occupation and clinical and demographic features. It enrolled 989 subjects who had undergone lumbar spine radiography.

The study of Goode et al.^29^ drew its subjects from a community study known as the Johnston County Osteoarthritis Project. Its sample was 1015 subjects who underwent lumbar spine radiography. The study of Ko et al.^30^ drew its subjects from patients who underwent CT scans for abdominal or urological disorders unrelated to back pain.

For the 2 studies by Kalichman et al.,^27^^,^^28^ subjects were drawn from the Framingham Heart Study. The sample was 138 subjects who had undergone CT scans screening for aortic calcification. The samples of the 2 studies strongly resemble one another, and appear to be the same except for 1 or 2 subjects.

For the study of Kjaer et al.^43^ subjects were drawn from the community of a County in Denmark, for a study that investigated the relationship between back pain and MRI findings. Osteoarthritis was not explicitly mentioned, but facet degeneration was graded using a 0-2 scale, with scores of 1 or 2 taken as indicating presence of facet degeneration. Most the data reported pertained to various changes in the intervertebral discs. In the published paper, data were reported on the numbers of subjects with facet degeneration who did or did not have pain, but comparative data on subjects without facet degeneration were not provided. However, the latter was available in an online supplement to a review of disc degeneration by Chou et al.^45^

Table 2 summarizes the data of the studies of lumbar Z joint osteoarthritis. It also provides the results of a number of statistical tests. These data can be evaluated in 2 ways. One is to follow the precepts of statistics. The other is to compare the data with those of other contexts.

**Table 2.** The data and statistical analysis of the population studies of lumbar Z joint osteoarthritis (OA).

	Source	ZJOA	Back pain		Prevalence pain		Prevalence OA		OR (95% CI)
			Yes	No	OA	No OA	Pain	No pain	
Radiographs	Lawrence^47^	Grade 2-4	101	75	57%	50%	20%	16%	1.4
		Grade 0-1	398	403	(50-64)	(46-48)	(17-24)	(13-19)	(1.0-1.9)
		Grade 3-4	19	15	56%	51%	4%	3%	1.2
		Grade 0-2	480	463	(39-71)	(48-54)	(3-6)	(2-5)	(0.6-2.4)
	Magora^46^	Present	142	110	56%	68%	38%	51%	0.6
		Absent	230	107	(50-62)	(63-73)	(33-43)	(44-57)	(0.4-0.8)
	Goode^29^	Present	247	239	51%	49%	59%	57%	1.1
		Absent	175	179	(46-55)	(44-55)	(54-63)	(52-62)	(0.8-1.4)
CT Scans	Ko^30^	Grade 2-3	33	50	40%	26%	25%	15%	1.9
		Grade 0-1	101	288	(30-51)	(22-31)	(18-33)	(11-19)	(1.2-3.1)
	Kalichman^27^	Grade 2-3	24	95	20%	20%	63%	63%	1.0
		Grade 0-1	14	55	(14-28)	(12-31)	(47-77)	(55-71)	(0.5-2.0
	Kalichman^28^	Grade 2-3	24	94	20%	18%	65%	63%	1.1
		Grade 0-1	13	56	(14-28)	(11-30)	(49-78)	(55-70)	(0.5-2.3)
MRI	Kjaer^43^	Slight/severe	106	46	70%	68%	37%	36%	1.1
		None	178	82	(62-76)	(63-74)	(32-43)	(28-45)	(0.7-1.6)

The figure in parentheses are the 95% CIs of the odds ratio (OR) or prevalence rates.

The aggregated data in Table 2 show that, in every study, the prevalence of Z joint osteoarthritis was not statistically greater in patients with pain than in patients with no pain. Nor did any study find osteoarthritis to be more common in patients with pain. These data preclude osteoarthritis, as seen on imaging, being incriminated as the cause of pain. Osteoarthritis of the lumbar Z joints is so common in asymptomatic individuals that seeing osteoarthritis on plain radiographs, CT scans, or MRI scans is no better than guessing, for distinguishing symptomatic from asymptomatic joints.

This lack of difference is borne out by the odds ratios. In all but one of the studies, the 95% CIs of the odds ratio overlap the value of 1.0. According to the precepts of statistics, this means that the observed ratios are not significantly different from values showing no association between osteoarthritis and pain. In the one study that is an exception, the CIs reached 1.1, which is barely >1.0. This indicates that the observed odds ratio (1.9) is only marginally significant statistically. This arises because although pain was more common in subjects with osteoarthritis (40%) than in those unaffected by osteoarthritis (26%), and although the prevalence of osteoarthritis in subjects with pain (25%) was greater than in subjects without pain (15%), in neither instance did the difference in prevalence reach statistical significance.

The second way of interpreting the data of Table 2 is to compare them with data on other joints. Some physicians might expect—or even argue—that osteoarthritis of the lumbar Z joints should be painful because osteoarthritis is a known cause of pain in other joints of the body. Table 3 summarizes the data on those other joints, which happen to be drawn from one of the studies examined in the present review.^47^

**Table 3.** A statistical analysis of the data on osteoarthritis (OA) of joints of the appendicular skeleton, from the population study of Lawrence et al.^47^

Joint	OA	Back pain		Prevalence pain		Prevalence OA		OR
		Yes	No	OA	No OA	Pain	No pain	(95% CI)
Hip	Grade 3-4	16	7	70%	8%	40%	2%	28.2
	Grade 0-1	24	296	(49-84)	(5-11)	(26-55)	(1-5)	(10-75)
Knee	Grade 3-4	39	18	68%	25%	13%	2%	6.7
	Grade 0-1	260	799	(56-79)	(22-27)	(10-17)	(1-3)	(4-12)
Proximal	Grade 3-4	33	58	36%	26%	25%	17%	1.6
Interphalangeal	Grade 0-1	101	288	(27-47)	(22-30)	(18-33)	(13-21)	(1.0-2.6)
Distal	Grade 3-4	24	95	20%	20%	63%	63%	1.0
Interphalangeal	Grade 0-1	14	55	(14-28)	(12-31)	(55-71)	(53-73)	(0.5-2.1
Metacarpo-	Grade 3-4	24	94	20%	15%	65%	63%	1.1
Phalangeal	Grade 0-1	13	56	(14-28)	(11-30)	(49-78)	(55-70)	(0.5-2.3)

Figures printed in parenthesis are the 95% CIs of the odds ratio (OR) or prevalence rate.

For large joints, such as the hip and the knee, the population data produce large odds ratios that dwarf those of Table 2. For these joints, pain is 3-9 times more common in subjects with osteoarthritis than in unaffected subjects, and the prevalence of osteoarthritis in subjects with pain is 7-20 times greater than its prevalence in subjects with no pain. The corresponding odds ratios are 28.2 and 6.7. So, there is no doubt that osteoarthritis is associated with pain.

Conspicuously, however, for proximal and distal interphalangeal joints and for metacarpophalangeal joints, the odds ratios of Table 3 are not significant, and closely resemble those of Table 2. For all 3 joints, the prevalence of pain does not differ between subjects with and without osteoarthritis, and the prevalence of osteoarthritis does not differ between subjects with and without pain.

So, if there is anything to question about osteoarthritis, it is not “why is lumbar Z joint osteoarthritis not painful when osteoarthritis of other joints is painful?” but “why is osteoarthritis painful in large joints but not in small joints such as the lumbar Z joints and the small joints of the hands?”.

The one anomalous population study of the lumbar Z joints was a study in which the authors reported a statistically significant but modest association between back pain and either severe osteoarthritis or severe osteoarthritis affecting 3 or more joints, in elderly patients.^31^ Their data do show statistically significant odds ratios (Table 4) but, in their elderly patients, neither the variable: “severe arthritis” nor the variable “severe arthritis at 3 or more joints” was significantly more common in patients with back pain than in patients with no pain; nor was pain significantly more common in patients with severe arthritis. This renders the odds ratios only marginally significant statistically. Moreover, whereas this association might apply to elderly patients with widespread, severe Z Joint OA, it cannot be extrapolated to the community in general; and it does not contradict the earlier conclusions by authors from the same institution.^27^^,^^28^^,^^33^

**Table 4.** Statistical analysis of the data of Suri et al.^31^ comparing back pain with the presence of severe osteoarthritis of the lumbar zygapophysial joints, or severe osteoarthritis affecting 3-8 joints.

Severe osteoarthritis	Back pain		Prevalence pain		Prevalence OA		OR (95% CI)
	Yes	No	OA	No OA	Pain	No pain	
Any	36	91	28%	17%	63%	47%	2.0
None	21	104	(21-36)	(11-24)	(50-74)	(40-54)	(1.1-3.6)
3-8 joints	20	36	36%	19%	35%	18.5%	2.4
<3 joints	37	159	(24-49)	(14-25)	24-48)	(13.6-24.5)	(1.2-4.6)

### Population studies cervical

Three population studies addressed the cervical Z joints. One was the study of mountain dwellers, described above in the context of lumbar Z joints.^39^ For cervical Z joints, this study had data on only 10 patients, which was too few to provide a meaningful statistical analysis.

The second population study was that of Lawrence et al.,^47^ described above, in the context of lumbar Z joints. It investigated 1787 patients with neck pain. The third study drew 5440 subjects from a large population study of chronic diseases in the Netherlands.^49^ In both the latter 2 studies, the subjects underwent plain radiography of the cervical spine. The data from these studies are summarized in Table 5.

**Table 5.** The data and statistical analysis of the 2 population studies of osteoarthritis (OA) of cervical zygapophysial joints (ZJ).

Source	ZJOA	Neck pain		Prevalence pain		Prevalence OA		OR (95% CI)	Age-adjusted OR (95% CI)
		Yes	No	OA	No OA	Pain	No pain		
Lawrence^47^	Grade 2-4	65	85	43%	38%	9%	8%	1.2	
	Grade 0-1	628	1009	(36-51)	(36-41)	(7-12)	6-10)	(0.9-1.7)	
	Grade 3-4	16	9	64%	38%	2%	1%	2.8	
	Grade 0-2	677	1085	(44-80)	(36-41)	(1-4)	(0-2)	(1.3-6.5)	
van der Donk^49^	Grade 2-4	49	184	21%	13%	7%	4%	1.7	
	Grade 0-1	701	4506	(16-27)	(13-14)	(5-9)	(3-5)	(1.2-2.4)	
Men	Grade 2-4							2.2	1.5
								(1.3-3.5)	(0.9-2.4)
Women	Grade 2-4							1.5	1.0
								(1.0-2.4)	(0.6-1.5)

The study of van der Donk^49^ did not provide the raw data for men and women or age.

The study of Lawrence et al.^47^ found no significant association between neck pain and grade 2-4 osteoarthritis of the cervical Z joints. For grade 3-4 osteoarthritis, the odds ratio is statistically significant, because osteoarthritis is twice as prevalent in patients with neck pain as it is in patients with no pain; but the actual prevalence rates are tiny (2% and 1%) and are not significantly different from one another statistically, which precludes any clinical significance to the data. Moreover, grade 3-4 osteoarthritis accounted for only 2% of the subjects with neck pain. However, another reservation applies.

The much larger study of van der Donk et al.^49^ also found a statistically significant odds ratio, and the CIs of the prevalence rates did not overlap. However, van der Donk et al.^49^ undertook a deeper, epidemiologically responsible analysis of their data. They found 2 features. One was that the crude odds ratios differed for men and women, with the odds ratio for women being not significant statistically while the odds ratio for men remained significant. The other feature was that the prevalence of osteoarthritis was strongly related to age, such that once odds ratios were adjusted for age they lost statistical significance, both for men and for women.

Notwithstanding these latter technical elaborations, even the crude odds ratios in Table 5 indicate only a marginal association between osteoarthritis and neck pain, with the prevalence of osteoarthritis being not significantly different, statistically, between subjects with or without neck pain.

### Diagnostic studies

The 4 diagnostic studies were each conducted in hospitals or practices that could perform imaging as well as diagnostic blocks. The objective of these studies was to test the utility of CT or MRI scans in predicting if Z joints affected by osteoarthritis were the source of pain. One study addressed cervical Z joints; the others addressed lumbar Z joints.

The cervical study^48^ reported that joints affected by higher grades of osteoarthritis were no more likely to respond to diagnostic blocks than joints with lower grades of osteoarthritis. However, the authors used a curious basis for this conclusion. They did not report how many patients obtained various degrees of relief of pain. They reported only the mean (standard deviation) relief reported by patients, which was 35%±41% when joints with osteoarthritis were blocked, and 33%±36% when unaffected joints were blocked. While these data might constitute circumstantial evidence that osteoarthritis of the cervical Z joints is not associated with pain, the ambiguity of the data precludes a definitive conclusion.

The older study of lumbar Z joints^44^ used a 0-12 scale to measure the severity of osteoarthritis in joints at the L3-4, L4-5, and L5-S1 levels, and performed placebo-controlled blocks. For their first block, patients were randomized to receive an active, intra-articular block using bupivacaine, or a control block using intramuscular normal saline. More than 50% relief of pain for at least 3 hours was regarded as a positive response. In each patient, blocks were performed separately and sequentially from L5-S1 to L4-5 and L3-4, until a source of pain was found or ruled out. Under this protocol, 316 blocks were performed across a total of 218 segmental levels. Subsequently, the scores for osteoarthritis were compared according to if pain was relieved or not when the target joint was anaesthetized. These comparisons were stratified by segmental level. The scores were compared using a Wilcoxon rank sum test. This test determines if there are significant differences between the numbers of joints with scores of different magnitudes (ranks) according to whether pain was relieved or not. This test found no statistically significant differences (Table 6).

**Table 6.** The comparison of median scores for osteoarthritis (OA) of the L3-4, L4-5, and L5-S1 zygapophysial joints, according to whether pain was relieved or not when the joint was anesthetized, based on the data of Schwarzer et al.^44^

Joint	Median score for OA (0-12)		
	Pain relieved		*P*-value
	Yes	No	
L3-4 Right	5.0	4.0	.25
L3-4 Left	5.0	2.0	.21
L4-5 Right	3.0	8.0	.19
L4-5 Left	1.0	8.0	.04
L5-S1 Right	7.0	7.0	.65
L5-S1 Left	5.5	6.0	.72

Although the scores differed for the left L4-5 joints, this was not shared by the right L4-5 joints.

Although this study was performed to test the predictive utility of CT scans, its data allow inferences to be drawn about the nature of osteoarthritis of the lumbar Z joints. On average, joints responsible for a patient’s back pain are not distinguishable on the basis of the severity of osteoarthritis affecting them. They exhibit a spectrum of severity that is not significantly different from the spectrum exhibited by joints not responsible for pain. Consequently, whether or not joints are responsible for a patient’s back pain is independent of the degree to which they are affected by osteoarthritis.

Some readers might consider that a limitation if this study is that positive responses were defined as at least 50% relief of pain, instead of complete relief. However, a companion study by the same investigators^52^ described the same sample of patients in a different context. Of the 23 patients who had relief of pain, 7 (30%) had complete relief of pain, and a further 11 (48%) had 90% relief. Therefore, the conclusions are not seriously compromised by the apparently generous definition of relief.

The second diagnostic study of lumbar Z joints^38^ investigated 50 patients. Osteoarthritis as seen on MRI was graded using a 0-3 scale. Joints were anesthetized using an intra-articular injection of 0.5 mL bupivacaine 0.25% and 0.5 mL triamcinolone. Comparison of the degree of relief from pain and the grade of osteoarthritis showed no statistically significant association (Table 7). A limitation of this study was that control blocks were not used; but this limitation is unlikely to adversely affect the results of the study, because those results were negative. Control blocks have the effect of decreasing the number of positive responses to blocks. This means that, had controls been used, even fewer cases of complete relief would have been found than reported in Table 7, making the prevalence of any positive relationship between osteoarthritis and pain absent or negligible.

**Table 7.** The results of the study of Hofmann et al.,^38^ showing the numbers of patients with and without osteoarthritis and their response to anesthetic blocks of the affected joint.

Osteoarthritis	Complete relief		Odds ratio
Grade	Yes	No	(95% CI)
2-3	4	26	0.4
0-1	3	17	(0.1-1.8)

The third diagnostic study^40^ investigated the utility of MRI in predicting if blocking a lumbar Z joint would relieve patients of their pain. Joints were classified as degenerated, hypertrophied, or both. They were anesthetized using medial branch blocks, which were controlled for false-positive responses by performing comparative blocks.^53^^,^^54^ Each joint was blocked on 2 occasions, with lidocaine or bupivacaine being used on the first occasion, and the opposite agent on the second occasion. A positive response was defined as >80% relief on both occasions, with the response to lidocaine lasting 1 h or more, and the response to bupivacaine lasting 3 h or more. Table 8 summarizes the results.

**Table 8.** Statistical analysis of the data of Stojanovic et al.^40^ that tested the association between degeneration or hypertrophy of a zygapophysial joint and relief of pain after performing comparative local anesthetic blocks of the affected joint.

Features of joint	80% relief after 2 blocks		Odds ratio
	Yes	No	(95% CI)
Degenerated and hypertrophy	13	56	0.5
Normal	11	22	(0.2-1.2)
Hypertrophy	11	23	1.0
Normal	11	22	(0.3-2.7)
Degenerated	3	34	0.2
Normal	11	22	(0.0-0.7)

One patient whose pain was relieved, and one whose pain was not relieved had joints that were both degenerated and hypertrophied, which explains why the totals of affected patients do not agree between the rows of the table.

Neither degeneration nor hypertrophy, alone or in combination, was associated with relief of pain. All odds ratios were ≤1.0. Of the patients who were relieved of pain, the proportion with affected joints (58%; 30%-78%) was not significantly different from the proportion not affected (42%; 22%-62%). Relief of pain occurred in only 24% of affected joints, compared with 33% of unaffected joints.

### Case-control studies

One case-control study provided evidence about lumbar Z joints.^41^ Two provided evidence about the cervical Z joints.^50^^,^^51^

The study of lumbar Z joints^41^ drew its data from a larger study of pain in the community. It recruited 162 subjects with chronic low back pain and 158 age-matched and gender-matched subjects with no pain. Osteoarthritis of the lumbar Z joints was graded 0-3, for none, mild, moderate, and severe. Table 9 summarizes the results.

**Table 9.** A statistical analysis of the data from the case-control study Hicks et al.^41^ that investigated the association between back pain and osteoarthritis of the lumbar zygapophysial joints.

Osteoarthritis	Segmental level								
	L3-4		OR (95% CI)	L4-5		OR (95% CI)	L5-S1		OR (95% CI)
	Pain	No pain		Pain	No pain		Pain	No pain	
Grade 1-3	110	88	1.7	155	148	1.5	160	157	0.5
Grade 0	52	70	(1.1-2.7)	7	10	(0.6-4.0)	2	1	(0.0-5.7)
Grade 2-3	8	5	1.6	30	32	0.9	55	46	1.2
Grade 0-1	154	153	(0.5-5.0)	132	126	(0.5-1.6)	107	112	(0.8-2.0)

Abbreviation: OR, odds ratio.

For osteoarthritis of any grade, there was a significant association at the L3-4 level, but not at L4-5 or L5-S1. For moderate to severe osteoarthritis, there was no association at any segmental level; the CIs of all the odds ratios overlapped 1.0. Although the association at L3-4 had a statistically significant odds ratio, the association was only marginal, because the prevalence of osteoarthritis in patients with pain (110/162; 68%; 61%-75%) was not significantly greater than in patients with no pain (88/158; 56%; 49%-64%).

In the earlier of the 2 case-control studies of cervical osteoarthritis,^50^ the authors reported the prevalence and distribution by segment of degenerative changes in the “posterior joints” in a sample of 160 asymptomatic subjects. For a comparison, they referred to data from one of their previous studies in which these same features were mapped in 100 symptomatic patients.^55^ The authors wrote that they found no differences in severity or segmental distribution of degenerative changes; but they did not provide the raw data upon which this comparison was made. Nonetheless, those data can be retrieved from their earlier publication.

**Figure 1.**
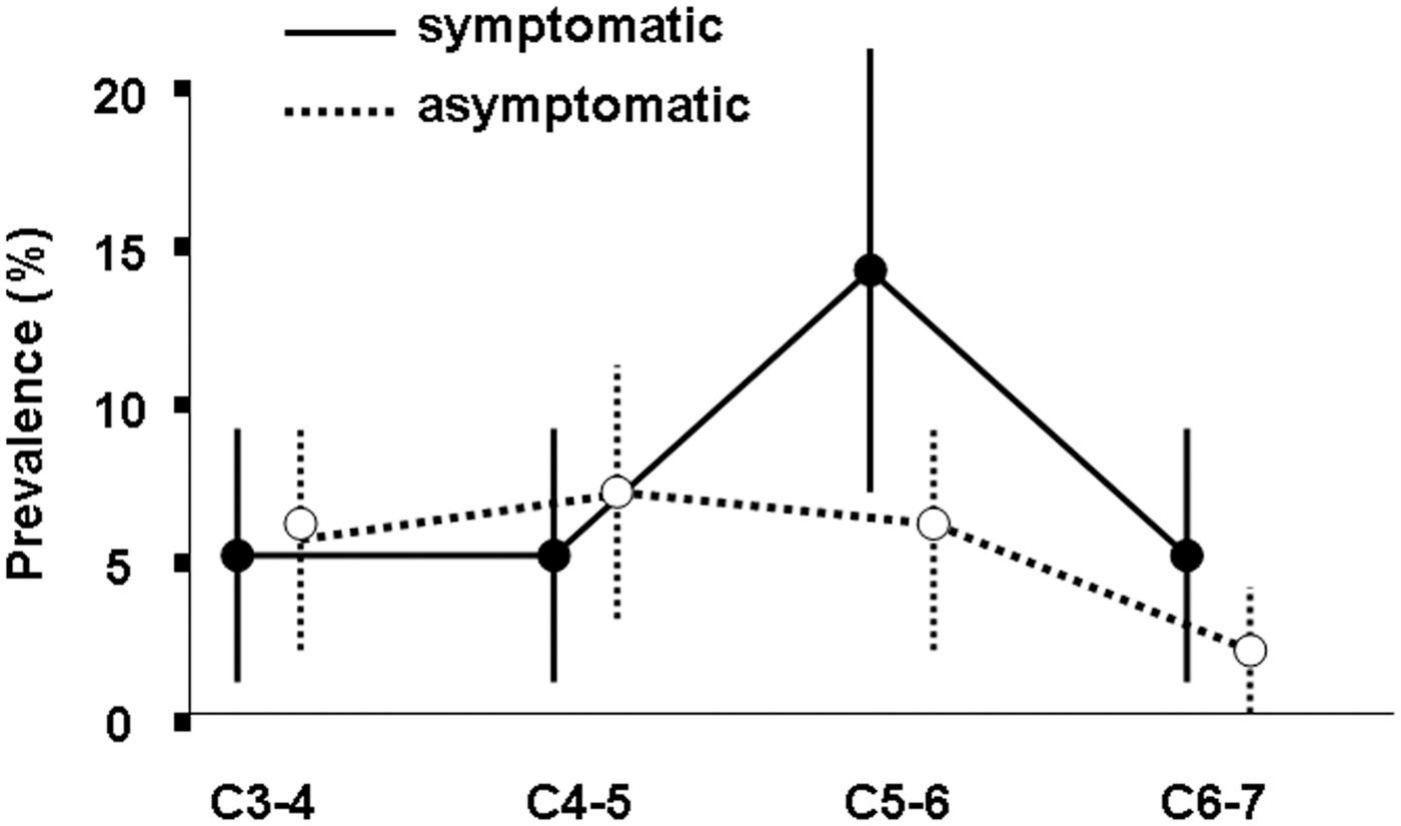
The data from Friedenberg and Miller^50^ and Friedenberg et al.^55^ comparing the prevalence by segment of degenerative changes in the cervical zygapophysial joints. The vertical bars indicate the 95% CIs of the respective prevalence estimates.

Figure 1 shows the prevalence of degenerative changes, by segment, in the symptomatic and asymptomatic subjects. At every segment, the 95% CIs of the prevalence estimates overlap, showing no significant differences in prevalence. By showing that osteoarthritis has a similar prevalence and distribution in symptomatic and asymptomatic patients, these data preclude invoking osteoarthritis as a general or common cause of neck pain. Osteoarthritis of the cervical Z joints is so common in the asymptomatic population that its presence on radiographs cannot distinguish an affected joint as one that is currently painful from one that has never been painful.

The second case-control study^51^ provides more decisive evidence. The study was conducted in the Radiology Department of a public hospital in the British health system. In addition to other features, this study identified “severe joint changes” in 653 patients referred for radiographs of the cervical spine because of neck pain, and in 365 patients with no neck pain who underwent cervical radiography in the course of “Barium studies.” The data obtained are summarized in Table 10.

**Table 10.** The association between neck pain and severe osteoarthritis (OA) of the cervical zygapophysial joints (Z joints), based on the data of Heller et al.^51^

OA	Neck pain		Prevalence pain		Prevalence OA		Odds ratio
Z joints	Yes	No	OA	No OA	Pain	No pain	(95% CI)
Severe	56	40	58%	65%	9%	11%	0.8
Other	597	325	(48-68)	(62-68)	(7-11)	(8-15)	(0.5-1.2)

Figures in parentheses are the 95% CIs of the odds ratio or prevalence rates.

Those data clearly show no significant association between osteoarthritis of the cervical Z joints and clinically significant neck pain. The odds ratio is <1.0, and the prevalence of osteoarthritis is not different between patients with pain and subjects with no pain. Neck pain is independent of the presence of osteoarthritis.

The study of Heller et al.^51^ also reinforced the observations in the population study of van der Donk^49^ (Table 5) that age is a confounding factor. Age is strongly associated with the presence of osteoarthritis both in symptomatic patients in subjects with no pain (Table 11). This makes osteoarthritis a normal age change unrelated to pain.

**Table 11.** The association between age and the presence of severe osteoarthritis of the cervical zygapophysial joints as seen on plain radiographs, in patients presenting with neck pain and subjects with no neck pain, according to the data reported by Heller et al.^51^

		Severe osteoarthritis		Odds ratio
Symptoms	Age	Yes	No	(95% CI)
Neck pain	≥60	43	156	9.4
	<60	13	441	(4.9-17.9)
Asymptomatic	≥60	34	109	6.6
	<60	10	212	(3.1-13.9)

### Gaps in evidence

A reasonable number of studies constitute the evidence-base on the association between osteoarthritis of Z joints and spinal pain. Seven population studies,^27–30^,43,46,47 3 diagnostic studies,^38^^,^^40^^,^^44^ and 1 case-control study^41^ have addressed the lumbar Z joints. Two population studies^47^^,^^49^ and 2 case-control studies,^50^^,^^51^ but no acceptable diagnostic studies have addressed the cervical Z joints. Notionally, this latter deficiency might constitute a gap in the evidence. However, there is a more fundamental gap: One that pertains to the approach to the question at hand, regardless of the method used.

Population studies have been the most commonly used method of studying osteoarthritis and spinal pain, because this has been the traditional method used for studying the joints of the appendicular skeleton. However, the population studies for spinal pain seem to have been undertaken with the expectation of finding a positive association between osteoarthritis and pain. They were not designed to *exclude* osteoarthritis as a cause of spinal pain.

When the results of population studies are positive and strong, they provide incriminating evidence of the cause of pain. However, when the results of population studies are negative they become contentious.

Population studies use various definitions of index cases, such as any pain at present,^49^ any pain in the last month^42^^,^^43^ or last year^39^^,^^42^ or ever.^47^ Regardless of the precise definition, the common feature is that, by and large, the pain is typically not of sufficient severity or persistence to warrant medical attention. This allows critics to claim that negative results from population studies do not exclude osteoarthritis being a cause of *clinically significant* spinal pain.

It is in this regard that diagnostic studies and case-control studies come to the fore. Respectively, these studies test if osteoarthritis occurs more commonly than not in Z joints proven to be the source of pain, and if osteoarthritis occurs significantly more often in patients with clinically significant pain than in asymptomatic subjects. In both instances, pain of sufficient severity to have warranted medical attention is the index condition.

Although more case-controlled studies would be welcome, their results would still only provide circumstantial evidence, because they would not show directly that Z joints affected by osteoarthritis were actually the source of pain. Case-control studies cannot exclude some other cause of pain that happens to be a covariate of osteoarthritis.

Only diagnostic studies provide direct evidence. However, in order for those studies to avoid becoming contentious, they would need to be prepared for negative results. They would need to be designed to reduce the risk of a Type II error, ie, having a negative result that is false-negative.

Diagnostic blocks would need to be rigorously controlled, and conducted on a double-blind basis, so that there is no doubt that the index joint(s) is, or is not, the source of pain. Meanwhile, in a manner akin to the requirements of noninferiority studies, the sample studied should be large enough that the CIs of measures such as odds ratios and prevalence rates leave no room for osteoarthritis being the cause of pain. Moreover, the sample should be large enough to cope with a low prevalence of positive responses to controlled blocks in the lumbar spine. The estimated prevalence of true lumbar Z joint pain (complete relief of pain) is only about 10%.^9^ This means that over 1000 patients would need to be studied in order to harvest 100 with complete relief of pain.

While these measures would secure the study against Type II errors, they would also secure the study if the results were unexpectedly positive. In order to overturn the consistently negative results of all the studies conducted to date, unexpected positive results would need to be strongly significant and beyond reproach.

Such studies would not be logistically difficult to conduct. Many clinics provide diagnostic blocks. Patients presenting to them are very likely already to have had imaging. All that then is required is for the clinic to perform rigorously controlled blocks; to maintain a record of the responses to those blocks; and to compare the images of those patients whose pain was completely relieved with the images of those not relieved. The cardinal requirement would be the patience needed to recruit the sufficiently large sample required, and to process the data.

## Discussion

It is not easy to evaluate empirically the validity and reliability of the search strategies used in a review article. For the present review, the paradigm criterion standard would be something like every article ever published on spine and pain. Applying filters in searches does not necessarily help. As illustrated by the present study, some relevant articles avoided discovery because they did not contain any conventional search terms.

Investigators who have studied osteoarthritis of the Z joints have not used the same terminology, either for the joint in question or the disease in question. Few studies used the official name of the joints, ie, “zygapophysial.” The American literature prefers the term: “facet,” but the British literature uses “apophyseal.” Others have used no name, instead referring to the Z joints as “posterior joints,” “articular processes,” or just “joints.” Likewise, synonyms for osteoarthritis include “arthritis,” “degeneration,” “degenerative changes,” or just “changes.” Any search that sought to be reliable would need to adjust to this diversity of language, by having wide filters.

Another feature encountered in the present study was the value of subject matter expertise. Studies not found by the searches conducted, and not cited in earlier publications, were nevertheless known to the present authors because of their long engagement in this corner of clinical practice. This expertise pertained not only to older studies that were not indexed or were poorly indexed in PubMed,^46^^,^^47^^,^^50^^,^^51^ but also to relevant data being located, with no prominence, in broader studies of degenerative changes focused principally on the intervertebral disc.^43^

Notwithstanding these technical issues, certain features suggest that the results of the present searches were reasonably thorough. The results of those searches agreed with the bibliographies of earlier studies and reviews, apart from 2 studies,^43^^,^^44^ which were nevertheless known to the present authors because of their subject matter expertise. Conversely, the present study discovered 4 studies^46^^,^^47^^,^^50^^,^^51^ that were not found either by the searches conducted or in the previous literature, and whose inclusion in the present review enriched the evidence-base.

“In any review, the conclusions drawn may—in principle—be affected by bias on the part of the authors of that review. In the present review, the data provided by the studies were so consistently and unambiguously negative that they are not subject to differences of opinion or differences in interpretation. Consequently, they leave little to no room for bias.”

With respect to the question addressed by the present review, 2 clear conclusions apply.

1. No study has produced any conclusive evidence that osteoarthritis is a cause of Z joint pain, either in the cervical spine or in the lumbar spine. Rather, the opposite applies. Although 2 population studies implied that there might be a marginal association between Z joint osteoarthritis and pain,^30^^,^^47^ 7 other population studies clearly found no such association.^27–28^,43,46,47,49 Furthermore, the diagnostic studies^38^^,^^40^^,^^44^ and the case-control studies,^41^^,^^50^^,^^51^ which studied clinically significant pain, consistently found no association.
2. Osteoarthritis of Z joints is so common in asymptomatic individuals that, even if it was a cause of pain, it could not be diagnosed with any confidence by any form of currently available medical imaging. The prevalence of osteoarthritis is essentially the same in subjects with pain as in subjects with no pain. Simply seeing osteoarthritis on images does not distinguish a painful joint from an asymptomatic one.

In technical terms, the studies to date have collectively failed to refute the null hypothesis that osteoarthritis of Z joints does not cause pain. For proof beyond doubt, the rigorous and large diagnostic studies described above under Gaps in Evidence would need to be completed.

Some physicians might believe that, despite this evidence, some Z joints affected by osteoarthritis might nevertheless be painful. This conjecture has prompted some investigators to search for biomarkers: Some feature, other than conventional imaging, that distinguishes painful joints from ones of similar appearance that are not painful. A variety of biomarkers have been explored to date, but only weak or inconsistent relationships have been found.

Patients with osteoarthritis of Z joints have slightly higher serum levels of hyaluronic acid than do patients with no arthritis or patients with disc degeneration or patients with disc degeneration as well as osteoarthritis,^56^ but elevated levels of hyaluronic acid are not associated with pain.^57^ Serum levels of Growth and Differentiation Factor 15 (GDF 15) and visceral adipose tissue-derived serine protease inhibitor (vaspin) are higher in patients with Z joint osteoarthritis, but their association with pain has not been tested.^58^ Levels of CXC Motif Chemokine Ligand 6 are somewhat higher in patients with back pain, and also in patients with Z joint osteoarthritis as well as back pain, but not to a degree that allows this biomarker to be in any way diagnostic^57^ of joints that have converted to being painful.

Although the pursuit of biomarkers might appear promising, until a definitive biomarker is found there is no way of identifying a joint with osteoarthritis that has converted from being asymptomatic to being painful. The contention that joints can do so remains no more than a conjecture.

A particular problem with idiom and language is the difficulty that some physicians and some insurers have in distinguishing between Z joint pain and Z joint osteoarthritis. Authors of teaching articles have conflated the 2 concepts. The following statements occur in the literature.

“Lumbar facet joint syndrome accounts for 15%-41% of patients with low back pain…and facet joint osteoarthritis is the most frequent form of facet joint syndrome.”^36^ “The lumbar facet joint constitutes ∼15%-45% of low back pain, with degenerative osteoarthritis as the most common form of facet joint pain.”^37^ “The most frequent cause of pain in facet joints is osteoarthritis.”^59^ “Facet joint degenerative osteoarthritis is the most frequent form of facet joint pain.”^60^ “…with ∼15%-25% of chronic back pain population suffering from lumbar facet arthropathy.”^61^ “Lumbar facet joint OA leads to localized lumbar pain, which may radiate unilaterally or bilaterally to the buttocks groin and thighs.”^62^

In all these examples, the authors attribute to Z joint osteoarthritis the properties of Z joint pain, or vice versa. This constitutes conflation. The prevalence figures cited in these examples all pertain to the prevalence of Z joint pain, which are different from the prevalence figures for Z joint osteoarthritis. None of those figures cited can apply to painful or symptomatic osteoarthritis, because no one has shown that osteoarthritis causes Z joint pain. Similarly, the referred pain patterns of lumbar Z joints cannot be represented as clinical features Z joint osteoarthritis. Those pain patterns were derived regardless of whether the stimulated joint had OA or not.

Conceptually, Z joint pain is a physiological diagnosis; it establishes the source of pain, without regard to its cause.^63^ The diagnosis is established using controlled, diagnostic blocks of the putatively painful joint. If the blocks consistently relieve the patient’s pain, its source has been found. Doing so distinguishes a source in a Z joint from other possible sources; and allows that particular pain to be treated specifically, by denervating the source, without regard to its cause.

The concept of Z joint pain should not be contaminated with illegitimate, vain attempts to include pathology in the diagnosis. Invoking osteoarthritis as the cause of pain is incompatible with the published evidence. Continuing to perpetuate views that are contrary to the evidence, either intentionally or by inattentive, casual writing, misrepresents that evidence to patients, insurers, and fellow physicians, and in some cases to Courts.

## References

[1] Speldewinde GC , Bashford GM, Davidson JR. Diagnostic cervical zygapophysial joint blocks for chronic cervical pain. Med J Aust. 2001;174(4):174-176. 11270757 10.5694/j.1326-5377.2001.tb143210.x

[2] Barnsley L , Lord SM, Wallis BJ, Bogduk N. The prevalence of chronic cervical zygapophysial joint pain after whiplash. Spine (Phila Pa 1976). 1995;20(1):20-25; discussion 26. 7709275 10.1097/00007632-199501000-00004

[3] Lord S , Barnsley L, Wallis BJ, Bogduk N. Chronic cervical zygapophysial joint pain after whiplash: a placebo-controlled prevalence study. Spine (Phila Pa 1976). 1996;21(15):1737-1745. 8855458 10.1097/00007632-199608010-00005

[4] Manchikanti L , Singh V, Rivera J, Pampati V. Prevalence of cervical facet joint pain in chronic neck pain. Pain Phys. 2002;3;5(7;3):243-249. 16902649

[5] Yin W , Bogduk N. The nature of neck pain in a private pain clinic in the United States. Pain Med. 2008;9(2):196-203. 18298702 10.1111/j.1526-4637.2007.00369.x

[6] Govind J , Bogduk N. Sources of cervicogenic headache among the upper cervical synovial joints. Pain Med. 2021;23(2):1-7. 10.1093/pm/pnaa469 33484154

[7] Schwarzer AC , Aprill CN, Derby R, Fortin J, Kine G, Bogduk N. Clinical features of patients with pain stemming from the lumbar zygapophysial joints. Is the lumbar facet syndrome a clinical entity? Spine (Phila Pa 1976). 1994;19(10):1132-1137. 8059268 10.1097/00007632-199405001-00006

[8] Manchikanti L , Pampati V, Fellows B, Bakhit CE. Prevalence of lumbar facet joint pain in chronic low back pain. Pain Phys. 1999;3;2(10;3):59-64. 16906217

[9] MacVicar J , MacVicar AM, Bogduk N. The prevalence of “pure” lumbar zygapophysial joint pain in patients with chronic low back pain. Pain Med. 2021;22(1):41-48. 33543264 10.1093/pm/pnaa383

[10] Engel A , King W, Schneider BJ, Duszynski B, Bogduk N. The effectiveness of cervical medial branch thermal radiofrequency neurotomy stratified by selection criteria: a systematic review of the literature. Pain Med. 2020;21(11):2726-2737. 32935126 10.1093/pm/pnaa219

[11] Schneider BJ , Doan L, Maes MK, Martinez KR, Gonzalez Cota A, Bogduk N; Standards Division of the Spine Intervention Society. Systematic review of the effectiveness of lumbar medial branch thermal radiofrequency neurotomy, stratified for diagnostic methods and procedural technique. Pain Med. 2020;21(6):1122-1141. 32040149 10.1093/pm/pnz349

[12] Yoon J , Efendy J, Redmond MJ. Septic arthritis of the lumbar facet joint. Case and literature review. J Clin Neurosci. 2020;71:299-303. 31843439 10.1016/j.jocn.2019.11.007

[13] Kitajima H , Hatano E, Kawaguchi M, et al Septic arthritis of cervical spine facet joints: a case report and review of imaging. Am J Case Rep. 2023;24:e941578. 37817401 10.12659/AJCR.941578 PMC10581355

[14] Konatalapalli RM , Demarco PJ, Jelinek JS, et al Gout in the axial skeleton. J Rheumatol. 2009;36(3):609-613. 19208604 10.3899/jrheum.080374

[15] Ben Tekaya A , Nacef L, Bellil M, et al Lumbar spinal involvement in calcium pyrophosphate dihydrate disease: a systematic literature review. Int J Gen Med. 2022;15:7639-7656. 36226310 10.2147/IJGM.S360714 PMC9550172

[16] Kobayashi T , Miyakoshi N, Abe T, et al Acute neck pain caused by pseudogout attack of calcified cervical yellow ligament: a case report. J Med Case Rep. 2016;10(1):133. 27237823 10.1186/s13256-016-0928-1 PMC4885118

[17] Motamedi K , Murphey MD, Fetsch JF, et al Villonodular synovitis (PVNS) of the spine. Skeletal Radiol. 2005;34(4):185-195. 15703944 10.1007/s00256-004-0880-9

[18] Zeoli T , Mathkour M, Scullen T, et al Spinal pigmented villonodular synovitis and tenosynovial giant cell tumor: a report of two cases and a comprehensive systematic review. Clin Neurol Neurosurg. 2021;202:106489. 33596487 10.1016/j.clineuro.2021.106489

[19] Chitta S , Russo TL, Albert AJ, Russo SS, MacFarlane JJ, Janish TJ. En bloc resection of cervical spine osteoid osteoma with O-arm-assisted 3D navigation: a case report. JBJS Case Connect. 2022;12(3):e21.00630. 10.2106/JBJS.CC.21.00630 36049033

[20] Kim JH , Sharan A, Cho W, Emam M, Hagen M, Kim SY. The prevalence of asymptomatic cervical and lumbar facet arthropathy: a computed tomography study. Asian Spine J. 2019;13(3):417-422. 30744307 10.31616/asj.2018.0235 PMC6547401

[21] Page MJ , McKenzie JE, Bossuyt PM, et al The PRISMA 2020 statement: an updated guideline for reporting systematic reviews. BMJ. 2021;372:n71. 33782057 10.1136/bmj.n71 PMC8005924

[22] Munn Z , Peters MDJ, Stern C, Tufanaru C, McArthur A, Aromataris E. Systematic review or scoping review? Guidance for authors when choosing between a systematic or scoping review approach. BMC Med Res Methodol. 2018;18(1):143. 30453902 10.1186/s12874-018-0611-x PMC6245623

[23] Tenny S , Hoffman MR. Odds ratio. In: StatPearls. StatPearls Publishing; 2024. https://www.ncbi.nlm.nih.gov/books/NBK431098/28613750

[24] Schofferman J. A narrative review of intra-articular corticosteroid injections for low back pain: Nikolai Bogduk. Pain Med. 2005;6(4):297-298. 16083459 10.1111/j.1526-4637.2005.00049.x

[25] Engel A , MacVicar J, Bogduk N. A philosophical foundation for diagnostic blocks, with criteria for their validation. Pain Med. 2014;15(6):998-1006. 24716821 10.1111/pme.12436

[26] Klessinger S , Bogduk N. Are false-positive rates of diagnostic medial branch blocks correct? Introducing the inconsistency rate. Interv Pain Med. 2023;2(4):100298. 10.1016/j.inpm.2023.100298 PMC11373007 39239224

[27] Kalichman L , Li L, Kim DH, et al Facet joint osteoarthritis and low back pain in the community-based population. Spine (Phila Pa 1976). 2008;33(23):2560-2565. 18923337 10.1097/BRS.0b013e318184ef95 PMC3021980

[28] Kalichman L , Kim DH, Li L, Guermazi A, Hunter DJ. Computed tomography-evaluated features of spinal degeneration: prevalence, intercorrelation, and association with self-reported low back pain. Spine J. 2010;10(3):200-208. 20006557 10.1016/j.spinee.2009.10.018 PMC3686273

[29] Goode AP , Marshall SW, Renner JB, et al Lumbar spine radiographic features and demographic, clinical, and radiographic knee, hip, and hand osteoarthritis. Arthritis Care Res (Hoboken). 2012;64(10):1536-1544. 22556059 10.1002/acr.21720 PMC3427717

[30] Ko S , Vaccaro AR, Lee S, Lee J, Chang H. The prevalence of lumbar spine facet joint osteoarthritis and its association with low back pain in selected Korean populations. Clin Orthop Surg. 2014;6(4):385-391. 25436061 10.4055/cios.2014.6.4.385 PMC4233216

[31] Suri P , Hunter DJ, Rainville J, Guermazi A, Katz JN. Presence and extent of severe facet joint osteoarthritis are associated with back pain in older adults. Osteoarthritis Cartilage. 2013;21(9):1199-1206. 23973131 10.1016/j.joca.2013.05.013 PMC4018241

[32] Kalichman L , Hunter DJ. Lumbar facet joint osteoarthritis: a review. Semin Arthritis Rheum. 2007;37(2):69-80. 17379279 10.1016/j.semarthrit.2007.01.007

[33] Gellhorn AC , Katz JN, Suri P. Osteoarthritis of the spine: the facet joint. Nat Rev Rheumatol. 2013;9(4):216-224. 23147891 10.1038/nrrheum.2012.199 PMC4012322

[34] Goode AP , Carey TS, Jordan JM. Low back pain and lumbar spine osteoarthritis: how are they related? Curr Rheumatol Rep. 2013;15(2):305. 23307577 10.1007/s11926-012-0305-z PMC3606549

[35] Raastad J , Reiman M, Coeytaux R, Ledbetter L, Goode AP. The association between lumbar spine radiographic features and low back pain: a systematic review and meta-analysis. Semin Arthritis Rheum. 2015;44(5):571-585. 25684125 10.1016/j.semarthrit.2014.10.006

[36] Du R , Xu G, Bai X, Li Z. Facet joint syndrome: pathophysiology, diagnosis, and treatment. J Pain Res. 2022;15:3689-3710. 36474960 10.2147/JPR.S389602 PMC9719706

[37] Alexander CE , Cascio MA, Varacallo M. Lumbosacral facet syndrome. In: StatPearls. StatPearls Publishing; 2023. 28722935

[38] Hofmann UK , Keller RL, Walter C, Mittag F. Predictability of the effects of facet joint infiltration in the degenerate lumbar spine when assessing MRI scans. J Orthop Surg Res. 2017;12(1):180. 29162138 10.1186/s13018-017-0685-x PMC5699022

[39] Tiwari P , Kaur H, Kaur H, Jha V, Singh N, Ashraf A. Prevalence of facet joint arthritis and its association with spinal pain in mountain population—a cross-sectional study. J Craniovertebr Junction Spine. 2020;11(1):36-45. 32549711 10.4103/jcvjs.JCVJS_121_19 PMC7274360

[40] Stojanovic MP , Sethee J, Mohiuddin M, et al MRI analysis of the lumbar spine: can it predict response to diagnostic and therapeutic facet procedures? Clin J Pain. 2010;26(2):110-115. 20090436 10.1097/AJP.0b013e3181b8cd4d

[41] Hicks GE , Morone N, Weiner DK. Degenerative lumbar disc and facet disease in older adults: prevalence and clinical correlates. Spine (Phila Pa 1976). 2009;34(12):1301-1306. 19455005 10.1097/BRS.0b013e3181a18263 PMC2867597

[42] Savage RA , Whitehouse G, Roberts N. The relationship between the magnetic resonance imaging appearance of the lumbar spine and low back pain, age, and occupation in males. Eur Spine J. 1997;6(2):106-114. 9209878 10.1007/BF01358742 PMC3454595

[43] Kjaer P , Leboeuf-Yde C, Korsholm L, Sorensen JS, Bendix T. Magnetic resonance imaging and low back pain in adults: a diagnostic imaging study of 40-year-old men and women. Spine (Phila Pa 1976). 2005;30(10):1173-1180. http://links.lww.com/BRS/A537 15897832 10.1097/01.brs.0000162396.97739.76

[44] Schwarzer AC , Wang S, O’Driscoll D, Harrington T, Bogduk N, Laurent R. The ability of computed tomography to identify a painful zygapophysial joint in patients with chronic low back pain. Spine (Phila Pa 1976). 1995;20(8):907-912. 7644955 10.1097/00007632-199504150-00005

[45] Chou D , Samartzis D, Bellabarba C, et al Degenerative magnetic resonance imaging changes in patients with chronic low back pain: a systematic review. Spine (Phila Pa 1976). 2011;36(21 Suppl):S43-S53. http://links.lww.com/BRS/A537 21952189 10.1097/BRS.0b013e31822ef700

[46] Magora A , Schwartz A. Relation between the low back pain syndrome and x-ray findings. I. Degenerative osteoarthritis. Scand J Rehab Med. 1976;8:115-125.

[47] Lawrence JS , Bremner JM, Bier F, Osteo A. Prevalence in the population and relationship between symptoms and x-ray changes. Ann Rheum Dis. 1966;25(1):1-24. 5905334 PMC2453365

[48] Hechelhammer L , Pfirrmann CWA, Zanetti M, Hodler J, Boos N, Schmid MR. Imaging findings predicting the outcome of cervical facet joint blocks. Eur Radiol. 2007;17(4):959-964. 17180331 10.1007/s00330-006-0379-y

[49] van der Donk J , Schouten JS, Passchier J, van Romunde LK, Valkenburg HA. The associations of neck pain with radiological abnormalities of the cervical spine and personality traits in a general population. J Rheumatol. 1991;18(12):1884-1889. 1795327

[50] Friedenberg ZB , Miller WT. Degenerative disc disease of the cervical spine. A comparative study of asymptomatic and symptomatic patients. J Bone Joint Surg Am. 1963;45(6):1171-1178. 14077981

[51] Heller CA , Stanley R, Lewis-Jones B, Heller RF. Value of x ray examinations of the cervical spine. Br Med J (Clin Res Ed). 1983;287(6401):1276-1278. 10.1136/bmj.287.6401.1276 PMC1549721 6416368

[52] Schwarzer AC , Wang S, Bogduk N, McNaught PJ, Laurent R. Prevalence and clinical features of lumbar zygapophysial joint pain: a study in an Australian population with chronic low back pain. Ann Rheum Dis. 1995;54(2):100-106. 7702395 10.1136/ard.54.2.100 PMC1005530

[53] Barnsley L , Lord S, Bogduk N. Comparative local anaesthetic blocks in the diagnosis of cervical zygapophysial joints pain. Pain. 1993;55(1):99-106. 8278215 10.1016/0304-3959(93)90189-V

[54] Lord SM , Barnsley L, Bogduk N. The utility of comparative local anaesthetic blocks versus placebo-controlled blocks for the diagnosis of cervical zygapophysial joint pain. Clin J Pain. 1995;11(3):208-213. 8535040 10.1097/00002508-199509000-00008

[55] Friedenberg ZB , Broder HA, Edeiken JE, Newton Spencer H. Degenerative disk disease of cervical spine. Clinical and roentgenographic study. JAMA. 1960;174:375-380. 13825054 10.1001/jama.1960.03030040029008

[56] Goode AP , Nelson AE, Kraus VB, Renner JB, Jordan JM. Biomarkers reflect differences in osteoarthritis phenotypes of the lumbar spine: the Johnston County Osteoarthritis Project. Osteoarthritis Cartilage. 2017;25(10):1672-1679. 28711584 10.1016/j.joca.2017.07.007 PMC5605465

[57] Goode AP , Hu D, George SZ, et al Biomarker clusters differentiate phenotypes of lumbar spine degeneration and low back pain: the Johnston County Osteoarthritis Project. Osteoarthr Cartil Open. 2022;4(3):100270. 35991624 10.1016/j.ocarto.2022.100270 PMC9387345

[58] Tarabeih N , Shalata A, Higla O, Kalinkovich A, Livshits G. The search for systemic biomarkers for monitoring degenerative lumbar spinal disorders. Osteoarthr Cartil Open. 2022;4(4):100323. 36601335 10.1016/j.ocarto.2022.100323 PMC9805972

[59] Anaya JEC , Coelho SRN, Taneja AK, Cardoso FN, Skaf AY, Aihara AY. Differential diagnosis of facet joint disorders. Radiographics. 2021;41(2):543-558. 33481690 10.1148/rg.2021200079

[60] Perolat R , Kastler A, Nicot B, et al Facet joint syndrome: from diagnosis to interventional management. Insights Imaging. 2018;9(5):773-789. 30090998 10.1007/s13244-018-0638-x PMC6206372

[61] Odonkor CA , Chen Y, Adekoya P, et al Inciting events association with lumbar facet joint pain. Anesth Analg. 2018;126(1):280-288. 28704245 10.1213/ANE.0000000000002242

[62] Abhishek A , Doherty M. Diagnosis and clinical presentation of osteoarthritis. Rheum Dis Clin North Am. 2013;39(1):45-66. 23312410 10.1016/j.rdc.2012.10.007

[63] Merskey H , Bogduk N, eds. Classification of chronic pain. In: Descriptions of Chronic Pain Syndromes and Definitions of Pain Terms. 2nd ed. IASP Press; 1994:108-109, 181-182.

